# 10 Minutes Frontal 40 Hz tACS—Effects on Working Memory Tested by Luck-Vogel Task

**DOI:** 10.3390/bs13010039

**Published:** 2022-12-31

**Authors:** Eugen Kvašňák, Eva Magyarová, Miroslav Domankuš, Michael Tesař, Jaroslava Kymplová, Vitaly Fetissov, Mohammed Abubaker, Wiam Al Qasem

**Affiliations:** 1 Department of Medical Biophysics and Informatics, Third Faculty of Medicine, Charles University, 100 00 Prague, Czech Republic; 2 Department of Psychiatry, First Faculty of Medicine, Charles University and General University Hospital, 121 08 Prague, Czech Republic; 3 Czech Institute of Informatics, Robotics, and Cybernetics, Czech Technical University, 160 00 Prague, Czech Republic; 4 Faculty of Biomedical Engineering, Czech Technical University, 272 01 Kladno, Czech Republic; 5 Faculty Hospital Královské Vinohrady, 100 00 Prague, Czech Republic

**Keywords:** working memory, transcranial alternating-current stimulation (tACS), EEG, reaction time, power spectral density, coherence, Luck–Vogel task

## Abstract

Working memory is a cognitive process that involves short-term active maintenance, flexible updating, and processing of goal- or task-relevant information. All frequency bands are involved in working memory. The activities of the theta and gamma frequency bands in the frontoparietal network are highly involved in working memory processes; theta oscillations play a role in the temporal organization of working memory items, and gamma oscillations influence the maintenance of information in working memory. Transcranial alternating current stimulation (tACS) results in frequency-specific modulation of endogenous oscillations and has shown promising results in cognitive neuroscience. The electrophysiological and behavioral changes induced by the modulation of endogenous gamma frequency in the prefrontal cortex using tACS have not been extensively studied in the context of working memory. Therefore, we aimed to investigate the effects of frontal gamma-tACS on working memory outcomes. We hypothesized that a 10-min gamma tACS administered over the frontal cortex would significantly improve working memory outcomes. Young healthy participants performed Luck–Vogel cognitive behavioral tasks with simultaneous pre- and post-intervention EEG recording (Sham versus 40 Hz tACS). Data from forty-one participants: sham (15 participants) and tACS (26 participants), were used for the statistical and behavioral analysis. The relative changes in behavioral outcomes and EEG due to the intervention were analyzed. The results show that tACS caused an increase in the power spectral density in the high beta and low gamma EEG bands and a decrease in left-right coherence. On the other hand, tACS had no significant effect on success rates and response times. Conclusion: 10 min of frontal 40 Hz tACS was not sufficient to produce detectable behavioral effects on working memory, whereas electrophysiological changes were evident. The limitations of the current stimulation protocol and future directions are discussed in detail in the following sections.

## 1. Introduction

### 1.1. Brain Oscillations and Working Memory

Brain (or neuronal) oscillations refer to the rhythmic and repetitive electrical activities of a large number of neuronal populations in the brain [1]. Neuronal oscillations are divided into five frequency bands: delta (0.5–3.5 Hz), theta (3.5–7 Hz), alpha (8–13 Hz), beta (18–25 Hz), and gamma (30–70 Hz), and they are involved in several functional processes in the brain [2,3]. Brain oscillations coordinate the dynamic interactions within and between brain regions involved in various stages of functional processes [4]. They can be detected using scalp electroencephalography (EEG), scalp magnetoencephalography (MEG), or intracranial EEG [5,6]. Cross-frequency coupling (CFC) refers to the interaction between brain oscillations in different frequency bands. CFCs are divided into six types: phase-to-amplitude, power-to-power, phase-to-phase, frequency-to-frequency, power-to- frequency, and phase-to-frequency interactions. The most common type of CFC is phase-to-amplitude coupling (PAC), in which high-frequency amplitudes are modulated by low-frequency phases [7,8,9,10,11,12]. The synchronization between the power and phase of fast and slow oscillations has been demonstrated in both the hippocampus and neocortical areas and is associated with several cognitive processes, including memory and attention [9]. The complex organization of neural activity is particularly important for cognitive processes. Abnormal interactions between brain oscillations have been reported in patients with neurodegenerative and neuropsychiatric disorders [13,14].

Working memory (WM) is a cognitive process that involves short-term active maintenance, flexible updating, and processing of goal- or task-relevant information (items, objectives, strategies) in a capacity-limited and interference-resistant manner [15,16]. WM is involved in higher cognitive processes, such as learning, reasoning, and mathematical skills [17,18]. To better understand the mechanisms underlying WM, several models have been developed [19,20,21]. Some of these models assume that the posterior parietal cortex is associated with the limited capacity of WM, while the frontal cortex is responsible for the executive functions and processing aspects of WM. In particular, the dorsolateral prefrontal cortex (DLPFC) supports both storage and processing functions of WM and maintains the memory of a sample trace in the presence of distractors [20,21,22]. The frontoparietal network is associated with mobile memory tasks [23]; in addition, phase synchrony between frontal and parietal cortices and the amplitude of theta frequency are related to coherent representations of mobile memory [24,25].

All brain oscillations are involved in the WM processing, particularly theta and gamma frequency bands [26,27]; theta oscillations play a role in the temporal organization of WM items, and gamma oscillations influence the maintenance of information in WM. The coupling between theta phase and gamma amplitude has been hypothesized as a mechanism underlying the WM process (theta/gamma neural code) [27,28]. In the context of the limited WM capacity and brain oscillations, two models have been adopted to understand the capacity-limited component of WM. The first model assumes that each gamma wave represents a single memory item and only a limited number of gamma waves can fit into a one theta cycle, thus limiting the capacity of WM [26,27]. The second model assumes that the entire gamma burst that fits into the theta cycle encodes for a single memory item [29,30]. During cognitively demanding tasks, endogenous gamma bursts nest in theta peaks in the frontal cortex (Peak-coupled CFC) [31].

### 1.2. Transcranial Electrical Stimulation

Transcranial electrical stimulation (tES) is a noninvasive brain stimulation technique that delivers weak electrical currents to the scalp [32]. tES is used to modulate endogenous brain activities to improve functional processes and/or interrupt pathological activities. Electrical stimulation of the brain can be delivered as a constant unidirectional current known as transcranial-direct current stimulation (tDCS), as a biphasic alternating current known as transcranial-alternating current stimulation (tACS), as a pulsed current known as transcranial-pulsed current stimulation (tPCS), or as electrical noise known as transcranial random noise stimulation (tRNS), in which a weak alternating current oscillating at random frequencies (typically 0.1 to 640 Hz) is delivered to the scalp [33]. The tES techniques have been studied in more than 70 neuropsychiatric conditions because of their simplicity, flexibility, and safety profile. Application areas include, but are not limited to, major depression [34], epilepsy [35], tinnitus [36], Parkinson’s disease (PK) [37], pain control [38,39], and stroke recovery [40,41]. tDCS and tACS are the most studied techniques in the field of cognition.

tDCS is thought to modulate resting membrane potentials and thereby alter spontaneous cortical activity. Unlike tACS, tDCS cannot be tailored to directly modulate specific brain network activity. In most studies, tDCS has been used in a polarity-specific manner, (i.e., whether the anode or cathode is placed on the defined cortical region results in an increase or decrease in activity) [42,43,44,45,46]. In general, anodal tDCS increases cortical excitability, whereas cathodal stimulation has the opposite effect [47]. However, many recent studies have revealed other mechanisms of tDCS beyond cortical excitability, as anodal tDCS can affect biological processes related to neuroprotection, and tDCS can also enhance cortical cholinergic activity, such as short-latency afferent inhibition [48,49,50]. In healthy subjects, tDCS has been shown to positively affect declarative memory [51], working memory [52], motor learning [53], verbal fluency [54], and planning ability [55,56].

tACS modulates cortical activity by affecting neuronal membrane potentials through oscillatory electrical stimulation at specific frequencies, thereby interacting with ongoing rhythmic cortical activity during cognitive processes [57,58]. tACS has frequency-specific effects on the brain dynamics, as measured by EEG [59,60,61] and by behavioral tasks. In general, the effects of tACS depend on the frequency of the applied alternating current [62,63]. tACS can be administered online or offline; offline tACS is administered immediately before or between tasks, whereas online tACS is applied during cognitive tasks. The “aftereffect” refers to the sustained brain activity that follows stimulation [64]. The aftereffect demonstrates changes in synaptic plasticity rather than entrainment *per se* [65]. Frequency-tuned tACS can improve vision [66,67], motor function [61,68,69,70,71], somato-sensitivity [70,72], cognitive processes, such as mobile memory [73,74,75], number discrimination ability [76], creativity [77], fluid intelligence, attention, and motor imagination [75,78]. Left frontal tACS appears to have a more pronounced effect on less cognitively demanding tasks, whereas left parietal tACS has an effect on more cognitively demanding tasks [79,80,81]. This is consistent with research showing that left-frontal tACS primarily affects the attentional components required for success on less cognitively demanding tasks [82].

Several studies have demonstrated tACS-induced EEG changes [60,79,83,84]. These changes can be summarized as follows; tACS applied to the subjects’ individual EEG alpha frequency resulted in an increase in EEG alpha amplitude, suggesting that this stimulation method can affect ongoing brain oscillations in a frequency-specific manner [60,83]. Pahor and Jaušovec showed that the theta-tACS stimulation increases theta power but has no effect on frequencies in the alpha range [82]. When the frequency of tACS overlaps with the native EEG spectrum, the oscillatory power coupled with the stimulation frequency may increase [60,69,72] and exhibit state-dependent effects [83,85].

In most tACS studies of cognitive function modulation, midband frequencies have been used across participants, e.g., 6 Hz for “theta-band stimulation” [86,87]. However, this approach produced inconsistent results, increasing the need for personalized frequency-specific tACS to enhance their efficacy in research and treatment [88,89]. For example, one could determine the individual theta frequency by identifying the theta band frequency with the highest power during the performance of a relevant task [90] or by relying on the theta-gamma frequency coupling, which requires identifying the theta band frequency that has the highest correlation with the gamma band frequency, which is usually achieved by quantifying the phase-amplitude coupling [91].

### 1.3. Transcranial Alternating Current Stimulation and Working Memory

#### 1.3.1. Theta-tACS

Theta oscillations play an essential role in local processing and functional connectivity [28]. Therefore, the cognitive effect of manipulating theta oscillations using tACS has been extensively studied [80,81,92,93,94,95]. Many studies have shown that the administration of theta-tACS over the frontal and/or parietal cortex leads to positive behavioral outcomes [80,81,92,95]. In contrast, Chander et al. (2016) showed that theta-tACS administered over the frontal midline impaired WM outcomes in the 2-back task [96], and Gonzalez-Perez et al. (2019) showed no cognitive enhancement effect of theta-tACS administered over the occipital cortex for perception and memory of facial and object stimuli [93]. In addition, Vosskuhl et al. (2015) stimulated a broad network of the fronto-parietal network at a frequency below the individual theta frequency to investigate the effect of such stimulation on memory performance; this protocol improved short-term memory capacity but not WM [97].

The synchronization of cortical oscillations in different frequency bands has been proposed as an important mechanism for high-level cognitive processes. The importance of phase synchronization (coherence) of native oscillations and tACS was demonstrated by several studies [75,98,99,100]. In-phase theta tACS between the right and left posterior parietal cortex or between the left prefrontal and posterior parietal cortex improved WM task outcomes and/or reaction times [75,99,100], whereas theta phase desynchronization in the frontoparietal region had a negative effect on WM task outcomes and reaction times [98]. Contrary to expectations, Kleinert et al. (2017) found no significant effects of theta-tACS (in-phase and anti-phase) applied over the right fronto-temporal regions on the results of the visuospatial WM tasks [101]. In-phase theta- tACS had no effects on EEG features, as suggested by Kleinert et al. (2017) and by (Alekseichuk et al., 2017) [98,101].

#### 1.3.2. Gamma-tACS

The analogous role of EEG gamma oscillation was demonstrated in the study of the modulation of EEG power and phase synchronization by auditory stimulation at beat frequencies corresponding to dominant EEG rhythms using intracranial recordings in preoperative epilepsy patients. Becher and colleagues found that the most striking increases in EEG power occurred after stimulation with 40 Hz monaural beats [102]. In healthy subjects, Santarnecchi et al. (2013) showed that gamma-band tACS administered over the left middle frontal gyrus reduced the time required to find the correct solution in a visuospatial abstract reasoning test similar to Raven’s matrices. They conclude that the WM load on the task is negligible. This represents a conceptual advance in our understanding of the neural signatures underlying fluid intelligence and is the first evidence for the causal involvement of high-frequency brain synchronization in human cognition, in contrast to views that consider gamma band activity merely as a by-product of neuronal activity [78,103,104].

Several studies investigated the effect of gamma-tACS on WM outcomes [105,106,107,108]. Hoy and colleagues indicated positive behavioral outcomes on high cognitive load tasks when gamma-tACS was applied over the prefrontal cortex [105]. Other studies showed no WM enhancement effect of gamma-tACS applied over the left middle frontal gyrus [109], fronto-parietal cortex [110], or occipital cortex [93]. In contrast, parietal gamma-tACS administered for 25 min/day for 5 days, in combination with cognitive training, significantly impaired WM training-related gains [107].

#### 1.3.3. Cross Frequency Coupling-tACS

The multiplexing-buffer model of WM assumes that short-term information is represented by the ordered activity of cell assemblies and that the multiple elements stored in WM are organized by theta-interleaved gamma subcycles [111]. The CFC-tACS protocols have been studied in several cognitive domains [112,113,114,115]. In the context of WM, Alekseichuk and colleagues [115] hypothesized that theta/gamma CFC in the prefrontal cortex plays an essential role in the WM process. They applied theta/gamma CFC-tACS to the left prefrontal cortex while performing a spatial WM task. They found that peak-coupled theta/gamma (gamma bursts above theta peaks significantly) tACS had a greater benefit on WM performance than theta tACS alone. The gamma frequencies associated with the optimal results were in the range of 80–100 Hz [115].

Based on the results of the studies summarized above, we hypothesized that brief (10 min) frontal exposure of healthy subjects to tACS at gamma frequency (40 Hz) will positively affect both behavior (e.g., reaction time, hit rate) and electrophysiological parameters (e.g., spectral features, coherence between right and left cortices) of the visual WM. We decided to stimulate the frontal cortex with gamma tACS because most studies stimulated the frontal cortex with theta tACS or the parietal cortex with gamma tACS. None of these studies examined the combined effect of frontal gamma-tACS on WM behavioral outcomes and electrophysiological coherence between the right and left cortices.

## 2. Material and Methods

### 2.1. Participants

Seventy-nine healthy, right-handed, non-colorblind young adult volunteers were recruited for the study after having given written informed consent. All participants have normal or corrected-to-normal vision, and all were right-handed, according to the Edinburgh handedness inventory. Participants were assigned into two groups (sham and tACS). None of the participants had symptoms or a history of psychiatric or neurological disorders, drug-dependent chronic diseases, or brain injury. All experiments were conducted in accordance with the Declaration of Helsinki and with the approval of the ethics committee of the Third Faculty of Medicine, Charles University in Prague.

### 2.2. Experimental Procedure

**Figure 1:**
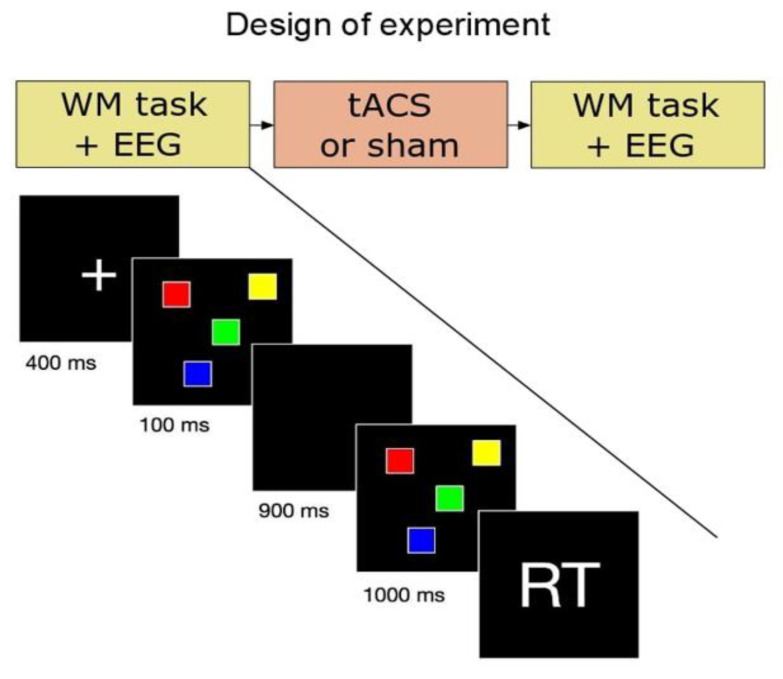
The Luck and Vogel paradigm (Luck and Vogel 1997). Each subject was exposed to 60 trials and each trial consisted of five screens. (1) A fixation dot to indicate the beginning of a new set of screens (400 ms); (2) the initial cue with the array of squares to memorize (100 ms); (3) a blank screen for memory retention (900 ms); (4) the target cue with the array of the same layout of squares as the initial cue but (in 50% of the trials) with one square color changed (1000 ms); (5) a blank screen for the subject response (duration: either until response or 6500 ms). Each array contained four squares with four of eight predefined colors (orange, grey, purple, red, blue, green, yellow, and white).

The experiment consisted of three consecutive parts (Figure 1) performed in one session. Every session consisted of three steps: (1) a behavioral task with a simultaneous EEG recording; (2) an intervention: 10-min tACS or sham stimulation (subjects were blinded only as to the type of intervention); (3) a behavioral task with simultaneous EEG recording after stimulation. All participants attended introductory sessions about the laboratory and the procedure.

### 2.3. Behavioral Task

Participants performed the visual WM task: the Luck and Vogel paradigm (Luck and Vogel 1997). The details of the task can be found in (Figure 1). During the introductory sessions, participants were allowed to do a few practice trials where the assistant ensured that the subjects understood the task before the experiments began. During the task, the subjects were asked to decide whether the squares in the initial and target cue were of the same colors or not. Responses were recorded by clicking on the left (“Yes”) or right (“No”) keys on a computer mouse. The experimental paradigm was fully implemented using OpenSesame 3.2 in Python 2.7 under Windows 7 SP2 64-bit. Participants were asked about their sensation during the tACS and sham stimulation to make sure that the participants were blinded to the type of the stimulation during the entire stimulation period (irritation, phosphenes, headache, and itching under the electrodes…etc.).

### 2.4. Electrophysiology

EEG recordings and electrical stimulation were performed using a Starstim^®^ wireless hybrid EEG/tCS 8-channel neurostimulator system with NIC 1.4.9 Software (Neuroelectrics Ltd.Barcelona, Spain) and a neoprene headcap. EEG was recorded using gel-filled electrodes at positions Fp1, Fp2, F7, and F8 (with the Cz electrode serving as a reference) using the 10–10 system. Electrode impedances were kept below 5 kΩ. Sponge stimulation electrodes (diameter 5 cm, moistened with 2 mL of physiological solution) were located at F3 and F4, with grounding at the earlobes. The intensity of stimulation AC was 1.5 mA peak-to-peak (5 ms RAMP in and 5 ms RAMP out) at a frequency of 40 Hz. To maintain the signal quality, electrode impedances were continuously checked, and participants were instructed to avoid blinking, swallowing, chewing, talking, and all possible expressions that could produce EEG artifacts.

### 2.5. Data Processing and Statistics

The reaction time (RT) and success rate (SR) were calculated. To avoid extreme values, the median RT was calculated rather than the average. RT is defined as the time span between the appearance of the screen with the target cue (start of the task, see Figure 1) and the correct response (mouse click). The percentage of correct answers during the behavioral task is defined as SR. The relative change of SR induced by the intervention (tACS or sham) is defined as (SR (after)–SR (before))/SR (before), where “before” and “after” refer to the intervention (tACS/sham). A statistical analysis was carried out on EEG epochs rather than subjects, and only for correct answers. RTs were processed for correct answer epochs < 3500 ms only. Median RTs before and after the intervention were calculated.

The linear model was used to simulate the effect of stimulation on RT. Based on the dataset with 14,681 epochs (indexed by subject and trial), a linear mixed model with interactions was constructed in R with a t-test with Satterthwaite’s method. EEG data were processed using EEGLAB 14.1.2 [116]. Since the data were recorded with the sampling rate 500 Hz in the proprietary format of the Starstim^®^, a custom plug-in was used to import the data from the manufacturer. For further analysis, the EEG data were high-pass filtered to 0.3 Hz and visually inspected for artifacts, such as eye blinks or muscle activity, and the affected segments were discarded. Consequently, frequencies outside the range of interest (>80 Hz) were excluded from further analysis and the data were re-referenced to the average. Power spectral density (PSD) for the correct answers before and after the intervention (tACS or sham) was calculated in the whole signal as a continuum where the frequencies out of interest (>80 Hz) were cut off.

In order to inspect the synchronization of the left and right frontal activity, the coherences Fp1–Fp2 and F7–F8 were computed from each correct answer signal segment as a minimum variance distortionless response (details in Banesty et al., 2005 [117]). To compare the effect of tACS, the relative change of coherence, as [C(after)–C(before)]/C(before), were calculated for both the sham and the tACS interventions.

## 3. Results

Data analysis was performed on 41 subjects: 21 females (age 18–22), 15 subjects in the sham group, and 26 subjects in the tACS group, because 27 subjects were excluded from the further analysis due to technical problems (salt bridges, noise, etc.); eight subjects were excluded due to heavy biological artifacts; and three subjects were also excluded due to incomplete data

### 3.1. Behavior

The success rate (SR) improved after sham by 3.4% and after tACS by 3.9% that correspond to simple learning only. There was a predictable effect of learning with t(77) = 9.13, *p* < 0.001 in between sessions without group interaction, so SR in the Luck–Vogel task before and after the intervention did not differ significantly t(77) = 1.57, *p* = 0.18. A *t*-test with the use of Satterthwaite’s method has been used to calculate behavioral statistics. There was no significant behavioral effect t(77) = −0.57, *p* = 0.57 of the 10-min-lasting tACS that we have applied during the study on working memory.

**Figure 2:**
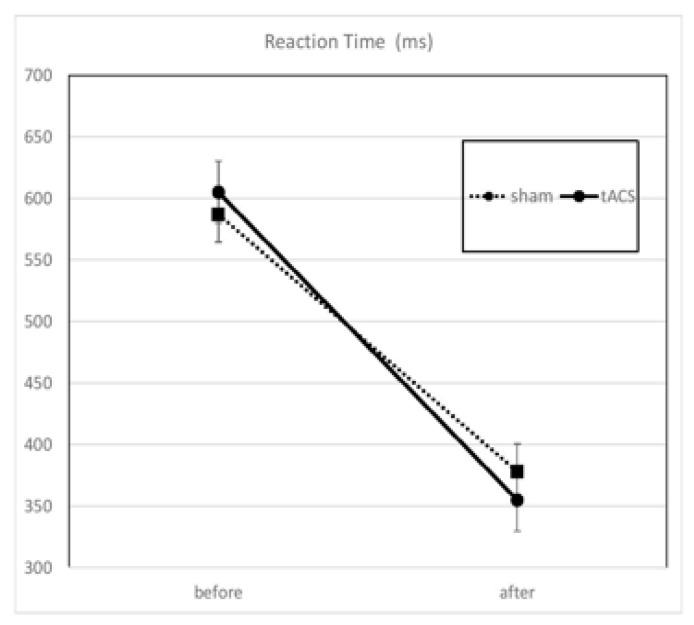
Reaction times in Luck-Vogel task before and after the intervention. The RTs were shortened in both groups post intervention, but the effect was not statistically significant.

The reaction times (RTs) to the Luck–Vogel task before and after the intervention were shortened in both groups (tACS or sham). On average, the subjects after tACS were faster by 23 ms compared to subjects after sham, but, considering the 17 ms difference between tACS and sham before the intervention, the final difference was not significant (Figure 2 and Table 1). The calculation of log (RT) showed that the 1st quartile was equal to −0.5762 and the 3rd quartile was equal to 0.5816.

### 3.2. Electrophysiology

**Figure 3:**
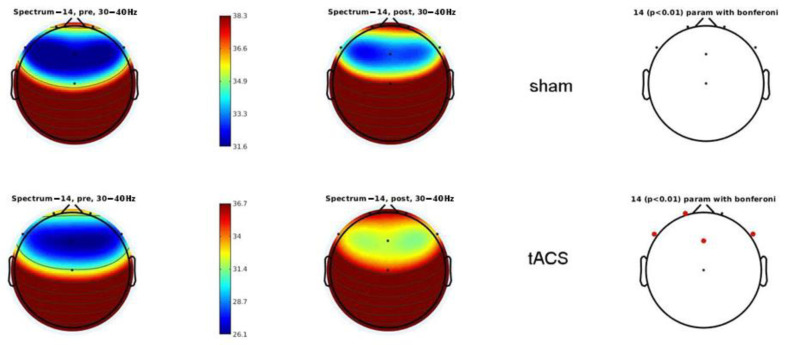
Power spectral density of low gamma activity at different electrodes in sham (upper pictures) and verum (lower pictures) tACS conditions. The left column shows pre-intervention, the middle column shows post–intervention, and the right column shows the comparison of the left and middle eons. Low gamma EEG activity differs at Fp1, F7, Fz, and F8 between pre- and “after” in the tACS group.

A power spectral density analysis revealed that the tACS group had significantly higher high beta (20–28 Hz) and low gamma (30–40 Hz) activities than the sham group. Compared to the sham group, high beta activity differs at F7, whereas low gamma EEG activity differs at Fp1, F7, Fz, and F8 in the tACS groups (Figure 3). These differences were observed throughout the recording session, whether the epochs were correct or not. No significant differences were observed at the other EEG frequency bands.

**Figure 4:**
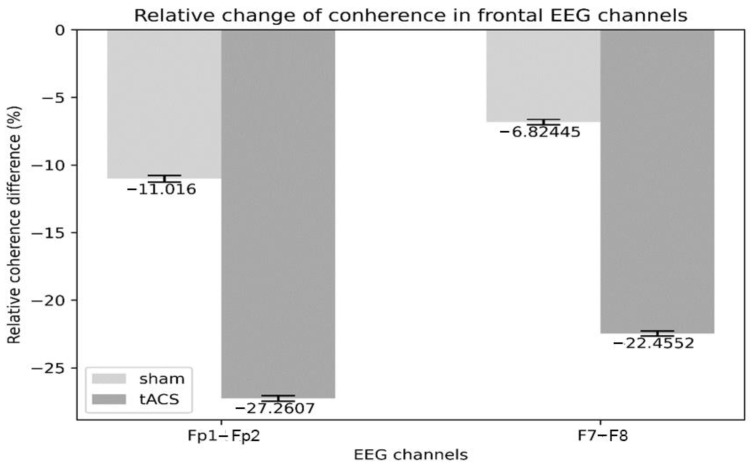
Relative change of EEG coherence of Fp1–Fp2 and of F7–F8.

Analysis of coherence for each trial showed a decrease after both interventions, but significantly more after tACS; Fp1-Fp2 coherence decreased by 27.3% after tACS, whereas it decreased by only 11.0% after sham. Similarly, F7–F8 coherence decreased by 22.5% after tACS, whereas it decreased by 6.8% after sham (Figure 4).

## 4. Discussion

Contrary to our expectations, the application of gamma-tACS over the frontal cortex did not affect the WM behavioral outcomes significantly. On the other hand, gamma-tACS elicited electrophysiological changes, especially at higher EEG frequencies. The behavioral and electrophysiological results of tACS are discussed in the following sections.

### 4.1. Behavior

In this study, a single session of 10 min −40 Hz- tACS administered over the prefrontal cortex in healthy subjects was not sufficient to boost WM. Compared to other studies in which gamma-tACS was administered over the DLPFC, Hoy et al. (2015) found that 40 Hz gamma-tACS administered for 20 min over the DLPFC selectively improved participants’ performance on higher cognitive load tasks (3-back task) [105]. On the other hand, Grover and colleagues administered gamma-tACS to the elderly for 20 min daily for four consecutive days and observed no behavioral effects of gamma-tACS on auditory–verbal WM, but found a significant effect on long-term memory [118]. In the current study, either the intensity of tACS was not high enough or the frequency of 40 Hz was not close to the eigenfrequency (to overlap with the Arnold tongue [119]) to demonstrate the behavioral effect. Furthermore, the results of the study demonstrated the Luck–Vogel cognitive task was not selective enough. This means that the task was either too cognitively demanding (e.g., more spots or more colors to remember, or a higher frequency of exposition) or too easy. Considering that the SR of the behavioral responses was 51%, which means that the behavioral phenomenon may be hidden by chance, this seems to support the idea of a too difficult task.

### 4.2. Electrophysiology

In this study, we investigated how gamma waves affect prefrontal cortex activity, with particular attention to WM. We found that gamma-tACS applied over frontal areas caused a significant increase in EEG activity at high beta and low gamma frequencies.

As Alekseichuk and colleagues showed, the amplification of high gamma oscillations during peaks or troughs of the theta wave would enhance or attenuate endogenous coupling, facilitating or impeding information processing in the affected brain area. They have shown that the simultaneous stimulation of theta and gamma waves in the prefrontal cortex enhances spatial WM only when repeated gamma bursts (particularly at 80–100 Hz) are phase-locked to the peaks of the theta rhythm.

This could imply that the tACS intervention has physiological effects on higher frequency bands associated with a cognitive task. Given that the subjects were subjected to a high-demand cognitive task, we would expect beta and higher frequencies to be more prominent, whereas alpha and lower frequencies would only occur as a result of fatigue. This phenomenon was observed in the tACS group, which demonstrated enhanced frontal lobe activity at higher frequencies. A more profound effect of gamma-tACS could be achieved by a finer individual tuning of the stimulation frequency (gamma-tACS), which may involve the gamma band, as suggested in some studies [67,120]. Spectral analysis showed that EEG activity increased in the narrow gamma band (37–40 Hz) after tACS compared to sham treatment, which could be attributed to an increase in cognitive activity. It is hypothesized that left-parietal tACS improved performance on difficult test tasks by increasing WM capacity, which correlates with studies on the relationship between WM processes and neural rhythms in frontal and parietal brain areas [79] and studies using tACS [80,81].

## 5. Conclusions, Limitations and Future Directions

Conclusion: A 10-min session of 40-Hz tACS administered to healthy young participants was not sufficient to produce detectable behavioral improvement in WM (as measured by the Luck–Vogel visual behavioral task), whereas this brief exposure to gamma tACS produced electrophysiological changes, evidenced by an increase in power spectral density in the high beta and low gamma EEG bands and a decrease in left-right coherence.

The current study is limited by the fact that a non-individualized fixed gamma frequency (40 Hz-tACS) was applied to all participants (Section 4.1) by the use of a between-subject design, and that the difficulty level of the Luck–Vogel task was set too high, and the baseline was too short (100 ms), so that the discrimination ability of the task fell below the required threshold.

In future studies, it is reasonable to (1) use a within-subject design because it eliminates the effects of differences in baseline characteristics on measured outcomes (for example, the same participant would receive the sham and verum in two sessions at least 72 h apart to eliminate the effects of stimulation from the previous session; the order of stimulation would be counterbalanced between participants); (2) construct a longer baseline of at least 500 ms, in the Luck and Vogel’s paradigm; (3) consider a different design of tACS, especially in terms of duration and the location of stimulation, personalized stimulation frequency (Section 1.2), and multiple sessions. It is known that a longer stimulation duration can lead to positive effects and that 20-min tACS stimulation is well tolerated by most subjects [113,121,122]. Therefore, the duration of the stimulation could be increased to 20 min in future studies; (4) other possible changes in the design of tACS would be the administration of daily tACS sessions on several consecutive days (e.g., 10 days of stimulation or 5 days/week for 4 weeks), as this strategy could produce a long-lasting, promising behavioral effect [123,124,125,126]; (5) the use of a peak-coupled theta/gamma tACS protocol may show promise for cognitive enhancement in future studies (Section 1.3.3); (6) to accurately examine WM phenomena in the high-frequency range, a high-frequency, high-density EEG setup must be used. The next study could be to replicate the here-presented experiment with Luck and Vogel’s paradigm on high-density EEG and check whether there are significant sources of signal in the DLPFC.

## Figures and Tables

**Table 1:** Reaction times in Luck–Vogel task before and after the intervention. The RTs were shortened in both groups post intervention, but the effect was not statistically significant. RT: reaction time; SD: standard deviation; SEM: standard error of the mean; tACS: transcranial-alternating current stimulation.

Condition	Median RT (ms)		SD		SEM	
	Before	After	Before	After	Before	After
Sham	587	378	244	139	39.6	22.5
tACS	605	355	255	162	40.3	25.3

## Data Availability

Data are available on request from the corresponding author.
